# The role of the precuneus in dissociative seizures: A structural neuroimaging study^☆^

**DOI:** 10.1016/j.nicl.2025.103872

**Published:** 2025-08-20

**Authors:** Leonie Helmstaedter, Stoyan Popkirov, Jörg Wellmer, Corinna Seliger, Johannes Jungilligens

**Affiliations:** a Department of Neurology, University Hospital Knappschaftskrankenhaus Bochum, Ruhr University Bochum, Bochum, Germany; b Department of Neurology and Center for Translational Neuro- and Behavioral Sciences (C-TNBS), University Hospital Essen, University Duisburg-Essen, Essen, Germany; c Ruhr Epileptology, Department of Neurology, University Hospital Knappschaftskrankenhaus Bochum, Ruhr University Bochum, Bochum, Germany

**Keywords:** Functional neurological disorder, Psychogenic nonepileptic seizures, Neuroimaging, Dissociation

## Abstract

The neurobiology and neuropsychology of dissociative seizures is increasingly well understood, but a gap remains in understanding the neurobiological underpinnings of one of its core features: dissociation. As a transdiagnostic phenomenon, dissociation has been related to alterations in precuneus function and structure in other neuropsychiatric disorders as well as through electrical stimulation. Still, the role of the precuneus in dissociative seizures needs further investigation. Here, we use retrospectively gathered neuroimaging data from 88 patients with dissociative seizures and 79 neurologically healthy controls to relate measures of illness severity and dissociative symptoms to cortical thickness and volume of the precuneus. Clinical-grade MRI scans from multiple scanners were processed using the FreeSurfer 7.4.1 recon-all-clinical pipeline and adjusted for scanner-related variability with the ComBat algorithm. No between-group differences were found; but in patients with dissociative seizures, older age at illness onset was associated with lower left and right precuneus volumes. Additionally, before correcting for multiple testing, we observed negative correlations between left precuneus volume and longer duration of illness, and between precuneus thickness and dissociative symptoms, which can inform future hypotheses. In conclusion, this study investigating the role of the precuneus in dissociative seizures found potential evidence for correlations of precuneus structure with clinical illness features and dissociative symptoms, albeit interpretations should be cautious due to the loss of significance through correction for multiple testing and the retrospective, exploratory nature of the study.

## Highlights

• First study specifically investigating the role of precuneus structure in adult dissociative seizure patients.
• Correlation between age at illness onset and lower left and right precuneus volumes.
• Potential relationship of precuneus structure with dissociative symptoms.

## 1 Introduction

Dissociative seizures – a subtype of functional neurological disorder also known as functional or psychogenic nonepileptic seizures − are episodes characterized by motor, emotional, perceptual, and autonomic-cognitive phenomena that might resemble epileptic seizures (Popkirov et al., 2019). Unlike epileptic seizures, dissociative seizures are defined as functional, indicating a lack of clear brain structural correlates of the seizure despite the obvious presence of symptoms. A core feature is the eponymous “dissociation”, which relates to the disintegration of psychological functions and involves alterations in consciousness and loss of control over cognitive and physical processes (Brown, 2016, Janet, 1889, Janet, 1894). Still, dissociation is one of the least well-understood aspects of dissociative seizures, dspite dissociative symptoms being closely related to higher seizure frequency, lower quality of life, higher rates of self-harm and suicide, and worse treatment outcomes (Campbell et al., 2022).

Dissociation as a transdiagnostic phenomenon does not only play a fundamental role in dissociative seizures but is also commonly present in patients with other psychiatric disorders, such as post-traumatic stress disorder, borderline personality disorder, and others (Krause-Utz et al., 2017, Roydeva et al., 2020). The pathophysiology of dissociation across these different disorders is considered to be similar to that of patients with dissociative seizures (Brown et al., 2007). While being incompletely understood, it is increasingly clear that the pathophysiology cannot be explained purely psychologically but involves functional and structural neurobiological features in cortical and subcortical structures (Krause-Utz et al., 2017, Roydeva et al., 2020). Similar evidence has been found in dissociative seizures, with studies identifying relationships between dissociative symptom severity and cortical volumes of bilateral insula, orbitofrontal, and cingulate gyrus (Jungilligens et al., 2022), and with functional connectivity in resting state networks such as the default mode network (van der Kruijs et al., 2014). The default mode network is a domain-general network related to internally focused mentation and disengagement/decoupling from external perception, and spans across several cortical nodes in the frontal, temporal and parietal lobes (Buckner et al., 2008). One of these nodes of the default mode network is the precuneus – a brain structure located in the posterior medial parietal cortex – which plays a key role in sense of body ownership, sense of agency, and the overall sense of a bodily self (Cavanna and Trimble, 2006, Harduf et al., 2023, Lyu et al., 2023).

**Fig. 1:**
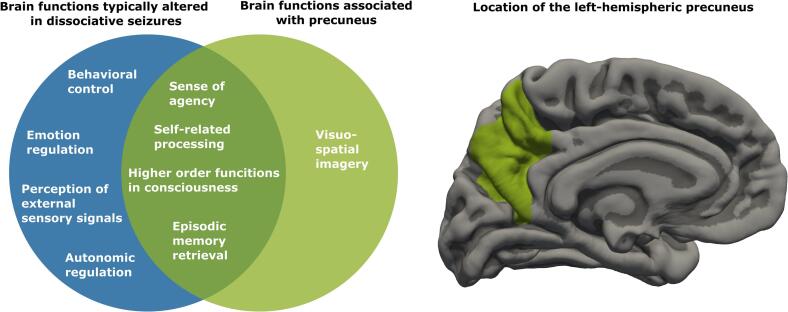
Overview on the overlap between brain functions that are typically altered in dissociative seizures (based on Reuber and Brown, 2017) and brain functions associated with the precuneus (based on Cavanna & Trimble 2006). The location of the precuneus in the left hemisphere is depicted based on the Desikan-Killiany cortical atlas (Desikan et al., 2006).

If disturbed, the breakdown of these high-level integrative processes can contribute to symptoms of bodily dissociation (e.g., feelings of detachment from oneself, feelings of derealization), as causal evidence from spontaneous epileptic seizures in the precuneus, and electric stimulation of the same region in epilepsy patients with depth-electrodes shows (Lyu et al., 2023, Parvizi et al., 2021, Vesuna et al., 2020). This relationship of precuneus function as a relevant node of the default mode network with sense of bodily self and – in case of disturbances – dissociative symptoms is further substantiated by findings from other neurologic and neuropsychiatric disorders (Dary et al., 2023). Beyond that, the precuneus as part of the default mode network has been examined in children and adolescents with functional neurological disorder, revealing overactivation in EEG and dysregulated neurometabolite concentrations (Kozlowska et al., 2018, Lan et al., 2025). Additional to functional studies, alterations in precuneus structure (gray matter volume and cortical thickness) are related to trait dissociation in children (Badura Brack et al., 2022), borderline personality disorder (Irle et al., 2007) and depersonalization disorder (Sierra et al., 2014). Thus, alterations in precuneus structure might be understood as predisposing to the development and maintenance of dissociative symptoms (Blanke et al., 2015, Cavanna and Trimble, 2006). However, findings on the role of the precuneus in neuropsychiatric conditions are heterogeneous, as alterations in structure and function may change across development due to the varying influence of stress (Kozlowska et al., 2017, Pervanidou and Chrousos, 2012, Trickett et al., 2010). Fig. 1 provides an overview on the overlap between functions associated with the precuneus (and general default mode network functions) and symptoms of dissociative seizures.

The involvement of the precuneus in dissociative symptoms is reflected in some initial findings from neuroimaging research in dissociative seizures. During an akinetic dissociative seizure incidentally captured during functional magnetic resonance imaging (MRI), deactivation in the right precuneus among other regions was recorded (Hologne et al., 2022). Additionally, Galucci-Neto et al. report group-level increases in activity during seizures in the right precuneus among others using single-photon emission computerized tomography in 26 patients with dissociative seizures (Gallucci-Neto et al., 2021). Our group previously reported altered transition probabilities between locus coeruleus-dependent brain states (co-activation patterns) that closely overlapped with the default mode network including the precuneus (Weber et al., 2024). In a different study, we identified correlations of brain structural markers with clinical features of dissociative seizures, where longer illness duration was related to lower cortical thickness of default mode network hubs including the precuneus (Zelinski et al., 2022). In a sample of patients with mixed functional neurological disorder including dissociative seizures, a recent study found a correlation between reduced cortical microstructural integrity in the left precuneus and left superior parietal cortex with functional physical symptom severity, additionally indicating a role of structural precuneus alterations in functional symptoms (Gninenko et al., 2025).

It is unknown, however, whether structural aspects of the precuneus are related to dissociative symptoms such as alterations in sense of a bodily self in dissociative seizures. Thus, in the current study, we investigated whether quantitative differences in cortical thickness and volume of the precuneus can be identified between patients with dissociative seizures and controls without macroscopic structural brain changes. In a second step, associations between structural characteristics and clinical features were examined. Considering the findings in other studies, we expect that cortical thickness and volume of the precuneus will correlate with clinical features such as illness duration and seizure duration, as well with dissociative seizure symptoms such as a more pronounced motor activity and disruptions of bodily self-perception.

This study’s approach is consistent with the considerations laid out by the Functional Neurological Disorders Society’s Neuroimaging Committee, which proposed detailed clinical symptom characterization and studies investigating the neural correlates of symptoms (Perez et al., 2021).

## 2 Methods

### 2.1 Participants

**Fig. 2:**
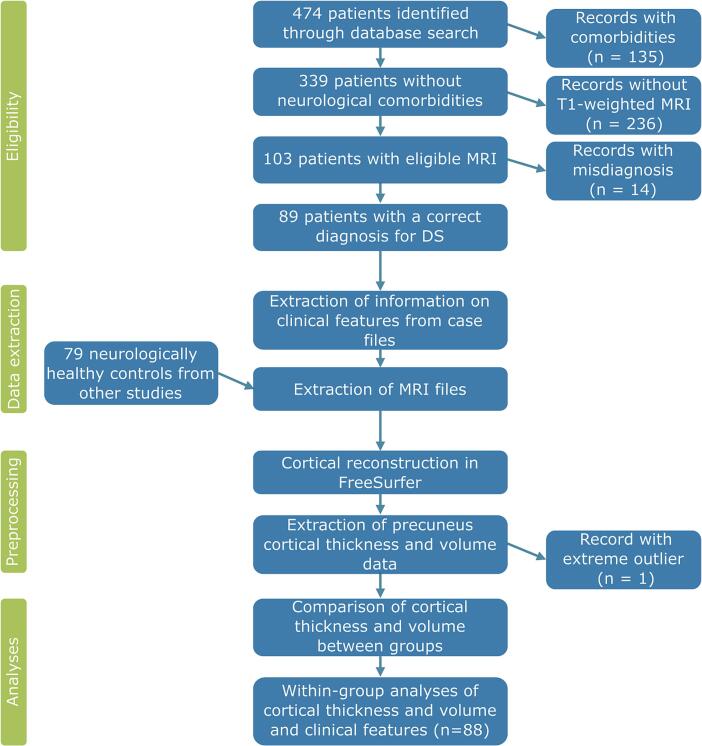
Overview on the inclusion and analysis workflow.

This research was approved by the ethics committee of the Medical Faculty, Ruhr University Bochum (reg.–no. 23–7915). In an initial retrospective search, we identified 474 patients with the diagnosis of dissociative seizures in the video-EEG database of the Ruhr Epileptology, a tertiary epilepsy center at the University Hospital Knappschaftskrankenhaus Bochum (Germany) using the diagnostic code F44.5. All cases were admitted between January 4th, 2010 (the opening of Ruhr Epileptology) and June 30th, 2024 (end of search). We then additionally applied inclusion criteria (diagnosis for “dissociative seizures”, video-EEG-confirmed or clinically established by at least two experiences epileptologists, at least one T1-weighted MRI scan) and exclusion criteria (co-morbid epilepsy or other neurological comorbidities with influence on the macrostructure of the brain, including development disorders or stroke). Fig. 2 depicts the participant selection process. The final group of patients consisted of 88 participants between the age of 18 and 66 (mean age = 36.1 years, SD = 13.7) of which 25 were male and 63 were female. A subset of these patients has been reported on in a previous study from our group (Zelinski et al., 2022). As a control group, we used data from neurologically and psychiatrically healthy controls (HCs; 34 participants without any established disease from other neuroimaging studies and 45 participants with syncope). Since no significant differences in the measures of interest emerged between these two subgroups, we treated them as one group of neurologically healthy controls.

### 2.2 Imaging data processing

All MRI data was visually inspected for quality issues such as movement artifacts before being exported for further preprocessing. To prepare cortical thickness and volume analyses, the FreeSurfer 7.4.1 recon-all-*clinical* pipeline was used. This new tool enables reliable performance of the FreeSurfer cortical reconstruction on clinical-grade MRI scans largely irrespective of imaging quality (Iglesias et al., 2023). It entails several extra procedures not part of the usual recon-all pipeline, including obtaining a volumetric segmentation and linear registration to Talairach space, synthetization of a higher resolution T1 image, and predicting the distance maps and reconstructing topologically accurate cortical surfaces. The other processing steps are equivalent to the standard recon-all pipeline and include automatic cortical reconstruction, surface inflation, registration to FreeSurfer’s default average spherical atlas, and parcellation (Fischl & Dale, 2000). To obtain cortical thickness measurements, the closest distance from the white/grey boundary to the grey/cerebrospinal fluid boundary was calculated individually at each point across the cortical mantle. Following this, the parcellation of the cortical surface in anatomically distinct regions was based on the Desikan-Killiany atlas. Outputs were visually inspected for quality and accuracy. Cortical thickness and cortical volumes values (individually for the left and right precuneus) as well as the FreeSurfer estimated total intracranial volumes and mean hemispheric thickness (to control for potential non-systematic influences of overall brain volume and thickness on our outcomes) were exported using the *aparcstats2table* command. All MRI machines that contributed data were research-grade 1.5 T or 3 T machines but mostly ran clinical scanning protocols since we relied on clinical data for this project. Because MRI data was obtained from different scanners and acquisition protocols (see Supplementary Table 1 for details), all cortical thickness and cortical volume data was corrected for scanner type additionally to age at MRI and sex by using the ComBat algorithm (Fortin et al., 2018) in neuroHarmonize (Pomponio et al., 2020). Age was included as a nonlinear term in the harmonization model (because we do not assume a linear relationship between age and brain structure), sex and scanner type were included as categorical variables.

### 2.3 Clinical data

In an exploratory approach, we extracted clinical information (duration of illness, age at illness onset, seizure frequency, typical seizure duration), and details about the specific symptoms of dissociation during the seizures from electronic casefiles, which included both information from individual patient anamnesis during take-in as well as data obtained from clinical examinations such as video-EEG and neuropsychological evaluations. Seizure frequency and duration were used as categorical variables (see Table 1 for categories). To characterize and quantify the diverse dissociative phenomena described in the casefiles, we relied on descriptive dissociation scores developed based on the Dissociation Tension Scale (Stiglmayr et al., 2010) and additional clinical observations. We created three different dissociation sub-scores by grouping symptoms as follows: 1) “Reduced perception of self and reality”: alterations in the sense of bodily self (self-location, body ownership, self-perspective, action capacity and perceptual body image), feelings of being disconnected from reality, and visual, olfactory or acoustic hallucinations during a dissociative seizure; 2) “Reduced consciousness and memory”: loss of consciousness during the seizure and amnesia regarding the seizure; 3) “Pain and sensory symptoms”: changes in pain and sensory functions during a seizure. As in previous publications (Cengiz et al., 2024) we classified the motor semiology of the seizures as either “major motor” (hyperkinetic or convulsive seizures); “minor motor” (motor signs are limited in spread and severity), and “akinetic” (syncope-like presentations) (Magaudda et al., 2016). Lastly, we extracted information about established current psychiatric comorbidities, which included depression, post-traumatic stress disorder, compulsive disorder and bipolar disorder.

**Table 1:** Overview on clinical features and categories.

	**M/SD**
**Clinical features**	
Age at illness onset	29.3 (14.8)
Duration of illness	6.9 (8.5)

	**N**
**Seizure frequency**	**69**
Daily	22
Weekly	20
Monthly	11
Yearly	7
Less than yearly	5
Once	4
*Information not available*	*19*

**Typical seizure duration**	**73**
<2min	24
<15 min	28
<30 min	7
>30 min	14
*Information not available*	*15*

**Reduced perception of self and reality**	**30**
Bodily self-perception	14
Sense of reality	6
Hallucinations	10
*Information not available*	*58*

**Pain and sensory symptoms**	**37**
Sensory	21
Pain	6
Both	10
*Information not available*	*51*

**Reduced consciousness and memory**	**53**
Memory	4
Consciousness	19
Both	30
*Information not available*	*35*

**Motor semiology**	**87**
Major motor	20
Minor motor	33
Atonic	34
*Information not available*	*1*

Due to retrospective information collection from case files, information was available for different quantities of subjects (designated by bold N in each category).

### 2.4 Statistics

SPSS was used to examine between- and within-group-differences individually in left and right precuneus volume and cortical thickness. ANCOVAs (controlling for estimated total intracranial volumes in case of volumetric analyses or mean hemispheric thickness in case of cortical thickness analyses) were conducted for the between-group analysis. For within-group analysis, we used partial correlations (controlling for estimated total intracranial volumes in case of volumetric analyses or mean hemispheric thickness in case of cortical thickness analyses) to examine the relationships between clinical features and precuneus cortical thickness and volume. In a second step, all analyses were additionally corrected for psychiatric comorbidities. The tests for duration of illness were conducted as two-tailed, while all other clinical disease features were tested one-tailed. Significance level was set at p < 0.05; correction for multiple testing/false discovery rate (FDR) was performed using the Benjamini-Hochberg method for each analysis (e.g., for illness duration) across the two measures (i.e., cortical volume and thickness) per hemisphere.

### 2.5 Control analyses

To understand the specificity of the findings related to the precuneus, we performed control analyses on the isthmus cingulate the, posterior cingulate (both part of the same large-scale brain network as the precuneus, the default mode network), and the lateral occipital cortex (not part of the same network). We perform these analyses within the same modality (i.e., if the original finding is related to precuneus cortical volume, we perform control analyses for cortical volumes of the control regions, but not for cortical thickness) but across hemispheres (i.e., if the original finding is related to the left precuneus, we perform control analyses for both the left-sided and the right-sided control regions).

## 3 Results

In total, 88 patients with dissociative seizures and 79 HCs were included. Table 1 presents an overview on the patient group.

ANCOVAs, either controlling for mean hemispheric thickness or total intracranial volume, showed no significant differences in left or right precuneus cortical thickness or volume between patients with dissociative seizures and neurological healthy controls (all p > 0.284).

**Fig. 3:**
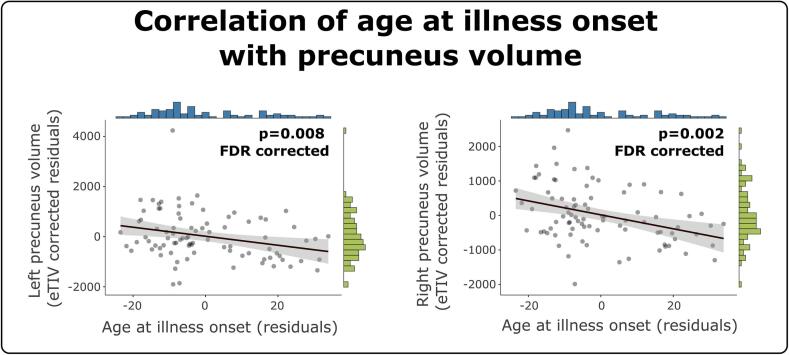
Correlation of age at illness onset with volumes of left and right precuneus (partial correlation, correcting for total intracranial volume, eTIV). Data on 88 patients included.

Using partial correlation correcting for total intracranial volume, we found a significant negative relationship between a longer duration of illness and cortical volume in the left precuneus (r = -0.195, p = 0.037 uncorrected; data on 88 patients available); but this did not survive FDR correction (p = 0.074 corrected). Additionally, age at illness onset and left and right precuneus volumes were negatively correlated, meaning that earlier illness onset was associated with higher volumes and later illness onset with lower volumes (left r = -0.282, p = 0.004 uncorrected; p = 0.008 FDR corrected; right r = -0.37, p = 0.001 uncorrected, p = 0.002 FDR corrected; data on 88 patients available; Fig. 3).

Regarding dissociative symptoms, partial correlation analyses (correcting for mean hemispheric thickness) showed that a higher score in the “Reduced perception of self and reality” score was associated with a reduced cortical thickness in the right precuneus (r = -0.183, p = 0.045 uncorrected; data on 30 patients available); this did not survive FDR correction (p = 0.075 corrected). The extent of pain and sensory symptoms correlated with decreased cortical thickness in the left precuneus (r = -0.191, p = 0.038 uncorrected; partial correlation correcting for mean hemispheric thickness; data on 37 patients available); this did not survive FDR correction (p = 0.076 corrected). No significant results were evident regarding seizure frequency (data on 69 patients available), seizure duration (data on 73 patients available) or the “Reduced consciousness and memory” score (data on 53 patients available). In order to analyze the relationship between motor semiology and precuneus volume, we conducted ANCOVAs, correcting for estimated total intracranial volume. The results showed a significant correlation between increased motor activity/movement and decreased cortical volume in the left precuneus (F (3, 82) = 2.82, p = 0.042 uncorrected; p = 0.084 corrected; data on 87 patients available). After correcting for psychiatric comorbidities, significant results remained unchanged. Control analyses to understand the specificity of the findings were performed on the isthmus cingulate the, posterior cingulate, and the lateral occipital cortex. All control analyses except one (significant negative relationship between duration of illness and cortical volume in the left isthmus cingulate; r = 0.213, p = 0.048 uncorrected) remained non-significant, speaking to the specificity of our findings. See supplementary material for details.

## 4 Discussion

Dissociation is a core feature of dissociative seizures, but its neurobiological underpinnings are not well-understood. Research from other neuropsychiatric disorders with overlapping symptoms and pathophysiology points to the precuneus as a critical node of the default mode network that might be related to the sense of bodily self and – if things go awry – to dissociative symptoms such as loss of body ownership and derealization. In this study, we used structural brain imaging data to assess the thickness and volume of the precuneus in patients with dissociative seizures, aiming to determine whether clinical illness features and retrospectively-determined dissociative seizure symptoms, based on chart documentation, are associated with these properties of brain structure. While we did not observe any differences in the cortical volume or thickness of the precuneus between patients with dissociative seizures and HCs, we did identify within-group variations related to age at illness onset and motor semiology. Other correlations with illness duration, seizure duration, seizure frequency and dissociative symptoms affecting the perception of self and reality, pain and sensory symptoms and consciousness and memory did not survive correction for multiple comparisons.

Contrary to our hypothesis, our results showed no differences between patients with dissociative seizures and neurological healthy subjects. However, this outcome is in line with previous studies that do not find group differences in structural measures between patients with dissociative seiz and healthy controls (Jungilligens et al., 2021, Zelinski et al., 2022). In the largest structural neuroimaging sample to date, Kerr and colleagues identified cortical thinning in superior temporal cortex and greater cortical thickness in the left lateral occipital cortex with an analysis approach comparable to our study (Kerr et al., 2022). Further, dissociative seizures and the associated symptoms and predisposing factors are vastly heterogeneous (Brown, 2006, Kuyk et al., 2008) and might potentially include clusters with different levels of involvement of specific brain network, thus contributing to heterogeneous findings across various studied populations. This within-group heterogeneity might also mask structural differences in the precuneus between groups (Popkirov et al., 2019).

Concordant with our hypothesis, we found correlations between precuneus thickness and volume with markers of illness severity and dissociative symptoms (reduced perception of self and reality, pain and sensory symptoms); with some not withstanding correction for multiple testing, indicating that these findings may be due to chance. We found a correlation between lower left and right precuneus volumes with older age at illness onset (surviving correction for multiple comparisons), indicating a larger precuneus volume in patients with a younger age at illness onset and smaller volume in patients with an older age at illness onset (despite correcting for age-effects!). This could be interpreted as a sign of a predisposing factor for the development of dissociative seizures. However, the relation between a longer duration of illness and a reduced cortical volume in the left precuneus, which is consistent with previous analyses (Zelinski et al., 2022) is somewhat contradictory to this and may suggest that cortical thinning in the precuneus develops over the course of illness rather than being a predisposition. Alterations in structure and function may vary throughout development (Kozlowska et al., 2017, Pervanidou and Chrousos, 2012, Trickett et al., 2010), which could contribute to these diverging findings.

As the findings regarding reduced perception of self and reality (self-localization, body ownership, first-person perspective, sense of agency, perceptual body image) do not survive correction for multiple comparisons, we can only cautiously interpret them as aligned with previous studies that linked the precuneus to the subjective perception of self and the disruption of this perception (Dary et al., 2023, Harduf et al., 2023, Lyu et al., 2023, Parvizi et al., 2021). Additionally, the control analyses on the isthmus cingulate the, the posterior cingulate, and the lateral occipital cortex remained non-significant, speaking to the specificity of our precuneus-related findings.

Integrating these findings in the larger field of functional neurological disorders yields important implications: Several resting state or task functional MRI studies found precuneus activity to be correlated to interoceptive processing of bodily signals from the functionally affected body part (Spagnolo et al., 2025), to failures in movements of the functionally affected limb (Cojan et al., 2009), to viewing negatively valenced emotional pictures in an emotion regulation task (Sojka et al., 2019), and to functional weakness symptom severity (Mueller et al., 2022). Our results cautiously add to these findings that relate alterations in precuneus structure to a breakdown in complex brain functions such as embodied consciousness and sense of agency. Importantly, we do not see potential functional or structural aberrations of the precuneus as sole predictors of functional neurological symptoms including dissociative seizures, but we do posit a role of the precuneus as one important region in the pathophysiology.

This study has several limitations, with a major one being the retrospective nature of the data collection, implying that the data was gathered from a clinical perspective and not specifically to address our research question. Therefore, some of the data we are investigating is incomplete. This includes, for example, that the patient case files that form the basis of our analyses were written from an epileptologists point of view (as patients mostly were seen in a differential diagnostics setting), meaning that the dissociative symptoms we are investigating were not necessarily the focus of the discharge letters. This is unfortunate especially since patients with dissociative seizures tend to have more difficulties describing details of their seizures compared to patients with epilepsy (Plug & Reuber, 2009), which complicates the evaluation of seizure descriptions. In our analysis we did not use validated psychometric instruments for dissociation, instead we created scores based on the dissociation tension scale. Additionally, given that this is a cross-sectional study, we cannot say if the clinical correlates we found in the precuneus are a predisposing factor or rather the consequence of ongoing dissociative seizure burden. Therefore, we were also not able to include the idea that the precuneus-volume and −thickness may develop throughout the course of aging. To control for this effect, future studies with larger cohorts could stratify participants according to age. This being said, the non-significant correlation between illness duration and precuneus volume or thickness suggests that the former is the case. Further, the MRIs we used for brain morphometry were performed on a range of different MRI machines for clinical purposes and not for the study itself; consequently, the MRIs do not necessarily live up to the standards of research-grade MRIs. To minimize the effect on our results, we used the FreeSurfer clinical preprocessing stream which was specifically developed and validated for this scenario and applied batch correction using the ComBat algorithm. The group of neurologically healthy controls included patients with syncope, for which we cannot rule out subtle influences of the syncopes on neurobiological factors. However, no significant differences in the measures of interest were found between patients with syncope and healthy controls. Finally, we cannot exclude that certain medications and therapies, such as psychotherapy, influenced the way patients experience their seizures or the probability of certain symptoms appearing during a seizure.

In conclusion, this first study specifically investigating the role of precuneus structure in adult dissociative seizure patients found potential evidence for correlations of precuneus structure with clinical illness features and dissociative symptoms, albeit interpretations should be cautious due to the loss of significance through correction for multiple testing and the retrospective nature of the study.

## Funding sources

SP is supported by a BMBF Advanced Clinician Scientist Programme UMEA^2^ (01EO2104). CS received intramural funding (registration number F1087-2024 and IF-038–24). This research is supported by intramural funding to JJ from FoRUM (registration number F1055-2022) and by the Sophia und Fritz-Heinemann-Stiftung.

## CRediT authorship contribution statement

**Leonie Helmstaedter:** Writing – original draft, Software, Investigation, Formal analysis, Data curation. **Stoyan Popkirov:** Writing – review & editing, Project administration, Methodology, Conceptualization. **Jörg Wellmer:** Writing – review & editing, Supervision, Resources, Project administration, Investigation. **Corinna Seliger:** Writing – review & editing, Resources, Funding acquisition. **Johannes Jungilligens:** Writing – original draft, Visualization, Validation, Supervision, Software, Project administration, Methodology, Formal analysis, Data curation, Conceptualization.

## Data Availability

The authors do not have permission to share data.
