# Supplementary material for: The role of the precuneus in dissociative seizures: A structural neuroimaging study
Source: Neuroimage Clin. 2025 Aug 20;48:103872. doi: 10.1016/j.nicl.2025.103872 (PMC12398941; doi:10.1016/j.nicl.2025.103872)
Supplement: Supplementary Data 1 [file mmc1.docx]

**Supplementary Material:**

**The Role of the Precuneus in Dissociative Seizures: A Structural Neuroimaging Study**

Leonie Helmstaedter, Stoyan Popkirov, Jörg Wellmer, Corinna Seliger, Johannes Jungilligens

**Control analyses for significant findings**

To understand the specificity of the findings related to the precuneus, we performed control analyses on the isthmus cingulate the, posterior cingulate (both part of the same large-scale brain network as the precuneus, the default mode network), and the lateral occipital cortex (not part of the same network). We perform these analyses within the same modality (i.e., if the original finding is related to precuneus cortical volume, we perform control analyses for cortical volumes of the control regions, but not for cortical thickness) but across hemispheres (i.e., if the original finding is related to the left precuneus, we perform control analyses for both the left-sided and the right-sided control regions).

**Original finding 1:** Using partial correlation correcting for total intracranial volume, we found a significant negative relationship between a longer duration of illness and cortical volume in the left precuneus (r = -0.195, p = 0.037 uncorrected).

**Control analysis 1:** Partial correlation between duration of illness and cortical volume of control regions:

|  | **Duration of illness** | |
| --- | --- | --- |
|  | **Pearson's r** | **p-value** |
| **lh_isthmuscingulate_volume** | 0.213 | 0.048* |
| **lh_lateraloccipital_volume** | 0.070 | 0.522 |
| **lh_posteriorcingulate_volume** | 0.041 | 0.703 |
| **rh_isthmuscingulate_volume** | 0.146 | 0.177 |
| **rh_lateraloccipital_volume** | 0.120 | 0.266 |
| **rh_posteriorcingulate_volume** | 0.006 | 0.957 |

*Note.* controlling for 'estimated total intracranial volume'; lh = left hemisphere, rh = right hemisphere.

**Original finding 2:** Additionally, age at illness onset and left and right precuneus volumes were negatively correlated (left r = -0.282, p = 0.004 uncorrected; p = 0.008 FDR corrected; right r = -0.37, p = 0.001 uncorrected, p = 0.002 FDR corrected).

**Control analysis 2:** Partial correlation between age at illness onset and cortical volume of control regions:

|  | **Age at illness onset** | |
| --- | --- | --- |
|  | **Pearson's r** | **p-value** |
| **lh_isthmuscingulate_volume** | 0.186 | 0.084 |
| **lh_lateraloccipital_volume** | 0.043 | 0.696 |
| **lh_posteriorcingulate_volume** | 0.006 | 0.955 |
| **rh_isthmuscingulate_volume** | 0.122 | 0.259 |
| **rh_lateraloccipital_volume** | 0.104 | 0.338 |
| **rh_posteriorcingulate_volume** | -0.021 | 0.843 |

*Note.* controlling for 'estimated total intracranial volume'; lh = left hemisphere, rh = right hemisphere.

**Original finding 3:** A higher score in the "Reduced perception of self and reality” score was associated with a reduced cortical thickness in the right precuneus (r = -0.183, p = 0.045 uncorrected.

**Control analysis 3:** Partial correlation between "Reduced perception of self and reality” score and cortical thickness of control regions:

|  | **Reduced perception of self and reality** | |
| --- | --- | --- |
|  | **Pearson's r** | **p-value** |
| **lh_isthmuscingulate_thickness** | 0.012 | 0.911 |
| **lh_lateraloccipital_thickness** | -0.210 | 0.051 |
| **lh_posteriorcingulate_thickness** | -0.020 | 0.856 |
| **rh_isthmuscingulate_thickness** | -0.062 | 0.571 |
| **rh_lateraloccipital_thickness** | -0.070 | 0.518 |
| **rh_posteriorcingulate_thickness** | -0.047 | 0.664 |

*Note.* controlling for 'lh_MeanThickness_thickness' *or 'rh_MeanThickness_thickness'*; lh = left hemisphere, rh = right hemisphere.

**Original finding 4:** The extent of pain and sensory symptoms correlated with decreased cortical thickness in the left precuneus (r = -0.191, p = 0.038 uncorrected; partial correlation correcting for mean hemispheric thickness).

**Control analysis 4:** Partial correlation between "Pain and sensory symptoms” score and cortical thickness of control regions:

|  | **Pain and sensory symptoms** | |
| --- | --- | --- |
|  | **Pearson's r** | **p-value** |
| **lh_isthmuscingulate_thickness** | 0.008 | 0.939 |
| **lh_lateraloccipital_thickness** | 0.054 | 0.622 |
| **lh_posteriorcingulate_thickness** | -0.197 | 0.068 |
| **rh_isthmuscingulate_thickness** | -0.082 | 0.452 |
| **rh_lateraloccipital_thickness** | -0.045 | 0.680 |
| **rh_posteriorcingulate_thickness** | -0.149 | 0.169 |

*Note.* controlling for 'lh_MeanThickness_thickness' *or 'rh_MeanThickness_thickness'*; lh = left hemisphere, rh = right hemisphere.

**Original finding 5:** An ANCOVA, correcting for estimated total intracranial volume, showed a significant correlation between increased motor activity/movement and decreased cortical volume in the left precuneus (F (3, 82) = 2.82, p = 0.042 uncorrected; p = 0.084 corrected).

**Control analysis 5:** ANCOVAs, correcting for estimated total intracranial volume, between motor activity/movement and cortical volumes of control regions:

- No significant correlation between increased motor activity/movement and decreased cortical volume left isthmus cingulate volume (F (3, 82) = 1.25, p = 0.297.
- No significant correlation between increased motor activity/movement and decreased cortical volume left lateral occipital corex volume (F (3, 82) = 1.43 p = 0.241
- No significant correlation between increased motor activity/movement and decreased cortical volume left posterior cingulate volume (F (3, 82) = 0.0681, p = 0.977.
- No significant correlation between increased motor activity/movement and decreased cortical volume right isthmus cingulate volume (F (3, 82) = 1.57, p = 0.203.
- No significant correlation between increased motor activity/movement and decreased cortical volume right lateral occipital cortex volume (F (3, 82) = 1.61, p = 0.193.
- No significant correlation between increased motor activity/movement and decreased cortical volume right lateral occipital volume (F (3, 82) = 0.472, p = 0.702.
