# Supplementary material for: The role of the precuneus in dissociative seizures: A structural neuroimaging study
Source: Neuroimage Clin. 2025 Aug 20;48:103872. doi: 10.1016/j.nicl.2025.103872 (PMC12398941; doi:10.1016/j.nicl.2025.103872)
Supplement: Supplementary Data 2 [file mmc2.docx]

| ***Supplementary Table 1.*** *Overview on magnetic resonance imaging scanners and acquisition protocols. Imaging data was preprocessed using the FreeSurfer 7.4.1 recon-all-clinical pipeline, which enables reliable cortical reconstruction on clinical-grade MRI scans largely irrespective of imaging quality.* | | | | | |
| --- | --- | --- | --- | --- | --- |
| **Scanner** | **Field strenght** | **Slice thickness** | **Echo time** | **No of patients** | **No of**  **healthy controls** |
| Achieva | 1.5 | 4 | 0.002176 | 1 | 0 |
| Aera | 1.5 | 1 | 0.00303 | 1 | 0 |
| Altea | 1.5 | 5 | 0.102 | 1 | 0 |
| Amira | 1.5 | 1 | 0.0032 | 1 | 0 |
| Avanto | 1.5 | 1 | 0.00281 | 10 | 0 |
| Avanto | 1.5 | 1.16 | 0.00419 | 0 | 8 |
| Avanto | 1.5 | 5 | 0.017 | 1 | 0 |
| Avanto | 1.5 | 6 | 0.0095 | 1 | 0 |
| Essenza | 1.5 | 5 | 0.011 | 1 | 0 |
| Harmony | 1 | 5 | 0.006 | 1 | 0 |
| Prisma | 3 | 0.85 | 0.00239 | 45 | 34 |
| Ingenia | 1.5 | 5 | 0.002409 | 1 | 0 |
| Panorama HFO | 1 | 1.8 | 0.006908 | 1 | 0 |
| Panorama HFO | 1 | 5 | 0.002109 | 1 | 11 |
| Prisma | 3 | 1 | 0.00229 | 12 | 0 |
| Prisma | 3 | 1.16 | 0.00438 | 0 | 1 |
| Prisma | 3 | 1.8 | 0.00239 | 0 | 25 |
| Prisma | 3 | 4 | 0.00249 | 2 | 0 |
| SIGNA Pioneer | 3 | 4 | 0.01 | 1 | 0 |
| Skyra | 3 | 0.9 | 0.00232 | 1 | 0 |
| Skyra | 3 | 4 | 0.009 | 1 | 0 |
| Sola | 1.5 | 1 | 0.00267 | 1 | 0 |
| Sola Fit | 1.5 | 5 | 0.108 | 1 | 0 |
| Titan3T | 3 | 3.5 | 0.04 | 1 | 0 |
| Verio | 3 | 4 | 0.00248 | 1 | 0 |
| Vida | 3 | 0.9 | 0.00232 | 2 | 0 |
